# Does Remote Monitoring Improve Time on Peritoneal Dialysis?: PRO

**DOI:** 10.34067/KID.0000001023

**Published:** 2025-12-05

**Authors:** Osama El Shamy

**Affiliations:** Renal, Electrolyte, and Hypertension Division, Hospital of the University of Pennsylvania, Perelman School of Medicine at the University of Pennsylvania, Philadelphia, Pennsylvania

**Keywords:** dialysis, peritoneal dialysis

Remote monitoring in peritoneal dialysis (PD) is one of the tools available to members of the care team to help troubleshoot, assist, and identify issues that can be interfering with the quality of dialysis treatment patients are receiving. Despite its many potential benefits, there has been some ambiguity regarding the implementation of protocols and utilization of the wealth of data that remote monitoring provides. In this article, I will review what constitutes remote monitoring, identify the data available through this technology, and explore the results of the available clinical studies. Remote monitoring has many benefits, some of which have been proven directly, others inferred, and finally a few that fall in the category of correlation rather than causality.

## Defining Remote Monitoring

There has been a spectrum of implementation protocols studied, all labeled as remote monitoring. From daily nurse assessments, direct interventions for missed treatments, system flagging protocols, to weekly clinical team evaluations and interventions, all have been studied under the guise of remote patient monitoring with varying results. This is perhaps the biggest weakness of remote monitoring and one that many of its detractors point out. The usefulness of any technology is almost always reliant on implementation, not on its mere existence. To date, there is no agreed-upon definition of what constitutes remote monitoring in PD.

## Remote Monitoring: Inception and Theory

One of the first reported implementations of remote monitoring was in Japan by Nakamoto *et al.*^1^ who developed a system to monitor elderly patients and patients with disability on automated PD (APD). The system consisted of two main components, a data collection system and a video-based support. The data collection system consisted of the APD cycler, automated BP measurements, and a scale. The video-based support system was a combination of a set-top box, a television, and a digital camera. This allowed providers to not only collect patients' clinical data but also provide support for patients with clear visualization of their home medical environment to assist with troubleshooting.

In the author's opinion, the theory behind remote monitoring goes beyond assisting elderly patients and patients with disability. The goal was to implement this technology in a manner that benefits all patients undergoing APD. Through remote monitoring, medical staff can identify patient treatment compliance, thereby providing an opportunity for interventions to increase patient prescription compliance. Early troubleshooting can also help identify issues that could have otherwise resulted in unplanned clinic and hospital visits. It can also help reduce the frequency of cycler replacement due to technical issues, thereby reducing interruptions in patient treatment delivery. Finally, knowing that cycler data are actively being collected and reported to the medical team and technical staff can help reduce the anxiety for patients and caregivers by providing their support staff the data needed to diagnose a variety of technical and medical issues that could be causing treatment interruptions.

## Remote Monitoring: Landscape and Clinical Studies

Remote monitoring efforts have been greatly enhanced by the advances in PD cycler capabilities. APD devices today report individual cycle fill and drain volumes and fill, dwell, and drain times. More advanced programs also calculate lost treatment times and lost dwell times on the basis of the APD prescription to which the cycler is programmed. Alarms are coded and recorded on the basis of the manufacturer's specific criteria, and some parameters may be amenable to readjustment by the clinical care team. All this information and more is available to members of the clinical care team and technical support staff to help troubleshoot treatment issues remotely *via* cloud-based data collection systems.

In a recent survey^2^ of 127 PD-providing nephrologists and nurses in the United States and Canada, 91% reported having access to a remote monitoring platform to access their patients' APD treatment data logs. However, only 31% of respondents reported having a standardized troubleshooting and response protocol to manage the various remote monitoring alarms and issues that arise. When asked about their perceived level of importance of having a standard protocol for remote data monitoring, the average response was 8 (on a scale of 0–10). There were also differences in the frequency of accessing patients' remote monitoring data, where most nurses were reviewing them multiple times a week and most nephrologists were reviewing the data during patients' monthly clinic visits.

Since the first reported remote monitoring APD study,^1^ there have been numerous studies exploring the effect of remote monitoring on patient outcomes. Many of these studies compared the remote monitoring patient cohort with a control group of patients on PD. The control group was often defined as the patients on traditional APD; however, we are aware of variations in what is considered traditional practice from one unit to another and from one country to another. Some of the reported findings of these studies include reduced hospitalization rates and hospital days^3,4^ and lower PD technique failure rates.^4^ Other studies found that patients in the remote monitoring group reported better sleep quality.^5^ These findings, although important, certainly do not imply causality because most of the beneficial outcomes can be seen with improved treatment compliance, a metric not reported in the aforementioned studies. However, increased time on therapy when using remote patient monitoring in APD was demonstrated in a matched cohort of patients on APD in Colombia.^6^ The use of remote patient monitoring resulted in statistically significant higher time on PD during the study period (18.95 versus 15.75 months, *P* < 0.001) over a 2-year period.^6^

A subsequent randomized controlled trial^7^ of approximately 800 patients on APD divided between traditional APD versus remote monitoring APD programs found that patients in the conventional APD group had higher all-cause mortality (subdistribution hazard ratio, 1.69; 95% confidence interval [CI], 1.39 to 2.05; *P* = 0.01) and higher cardiovascular deaths (subdistribution hazard ratio, 2.44; 95% CI, 1.72 to 3.45; *P* < 0.001). Furthermore, they had higher hospitalization rates for fluid overload and insufficient dialysis (incidence rate ratio, 3.24; 95% CI, 1.24 to 7.53; *P* = 0.03) and reached the composite end point of cardiovascular mortality, first adverse event and hospitalizations related to cardiovascular disease, fluid overload, and insufficient dialysis efficiency approximately 26 days sooner than the remote monitoring (RM) group.^7^

It, therefore, comes as little surprise that remote monitoring APD reduced overall health care costs in both Colombia and Poland owing to the fewer hospitalizations, shorter lengths of hospital stay, greater number of months free of complications, and fewer episodes of peritonitis seen in the remote patient monitoring cohort resulted.^8,9^

## Proactive Care and Shortened Clinic Visits

In the aforementioned randomized crossover trial^6^ of 15 patients on PD, a significantly higher number of modifications were made to the APD prescriptions when they were in the RM portion of the study (60% versus 7%, *P* = 0.01). Subsequently, they had significantly shorter regular clinic visits (813 versus 1024 minutes, *P* < 0.001) and a reduced total amount of health care resource consumption (0.80±1.32 versus 1.87±2.39 times/12 weeks, *P* = 0.02). Moreover, in another study,^7^ the rate of identification of device mechanical complications was higher in the RM group (incidence rate ratio, 0.41; 95% CI, 0.22 to 0.66; *P* = 0.001). This allowed for timely interventions to reduce interruptions in patient treatment delivery.

## Patient and Clinician Perceptions of Remote Monitoring

When randomly assigned to receive APD with and without remote monitoring, patients assigned to the remote monitoring group reported statistically significant higher effectiveness (dialysis ability to relieve symptoms, prevent or treat condition, and amount of time it takes for dialysis to start working) and convenience (ease of use, dialysis planning, convenience) scores.^10^ Moreover, reduced health care resource consumption and shorter monthly clinic visit times were seen in the remote monitoring cohort.

The availability of and access to more complete data remotely reduces the burden of logbook noting of clinical data traditionally assigned to patients. It can also reduce patient-initiated communications because clinical staff can identify complications that patients might not be aware of. However, potential drawbacks include inadequate technologic literacy affecting treatment implementation and delivery, especially for older and underprivileged patients. The transfer of treatment data remotely also requires broadband access, which could be an issue for some patients. The accessibility of remote monitoring by the clinical staff also raises the questions of accountability and responsibility. Clinical staff are overworked as it is; adding remote monitoring data evaluation and troubleshooting to their list of responsibilities risks further exacerbating the issue.

## Conclusion

Remote patient monitoring provides clinical support staff with extensive treatment information that can provide the opportunity to not only assist with troubleshooting tasks but also proactively address clinical issues as they develop before they become significant. Such issues include prescription noncompliance and prolonged fill and/or drain times. Most of the nephrologists and nurses in North America have access to remote monitoring data and are in favor of the implementation of a standardized protocol to help guide treatment decisions. Numerous studies have been published that demonstrated increased time on PD, reduced hospitalizations, reduced hospital days, lower all-cause mortality, lower cardiovascular mortality, higher patient satisfaction scores, and the cost-effectiveness of remote monitoring in APD.

Correlation does not prove causality, but it is not inconceivable that access to patients' treatment logs and missed treatment data, guided by a protocolized assessment and intervention approach, can improve time on PD, as demonstrated by Sanabria *et al*.^6^ The technology is here, and the time is now. It is incumbent on the clinical staff, manufacturers, and patients to work together to use this technology in the most efficient and beneficial way for patient care.
